# Existence of solutions for stress-rate type strain-limiting viscoelasticity in Gevrey spaces

**DOI:** 10.1098/rsta.2022.0374

**Published:** 2023-12-25

**Authors:** L. Bachmann, F. De Anna, A. Schlömerkemper, Y. Şengül

**Affiliations:** ^1^ Institute of Mathematics, University of Würzburg, Emil-Fischer-Straße 40, 97074 Würzburg, Germany; ^2^ School of Mathematics, Cardiff University, Cardiff CF24 4AG, UK

**Keywords:** viscoelasticity, stress-rate model, strain-limiting theory, stress equation, local smooth solutions, Gevrey classes, Sobolev inflation

## Abstract

In this work, we deal with a one-dimensional stress-rate type model for the response of viscoelastic materials, in relation to the strain-limiting theory. The model is based on a constitutive relation of stress-rate type. Unlike classical models in elasticity, the unknown of the model under consideration is uniquely the stress, avoiding the use of the deformation. Here, we treat the case of periodic boundary conditions for a linearized model. We determine an optimal function space that ensures the local existence of solutions to the linearized model around certain steady states. This optimal space is known as the Gevrey-class 3/2, which characterizes the regularity properties of the solutions. The exponent 3/2 in the Gevrey-class reflects the specific dispersion properties of the equation itself.

This article is part of the theme issue ‘Foundational issues, analysis and geometry in continuum mechanics’.

## 1. Introduction

Implicit constitutive theory, which has been of great interest recently, provides useful tools to approach a collection of models describing response of materials [1,2]. Among other advantages, it leads to a different small strain theory allowing for a nonlinear relationship between the linearized strain and the stress. The main aim of this work is to investigate such a strain-limiting model for the response of vicoelastic materials. More precisely, we are interested in studying a model where the linearized strain is given as a function of the stress and the stress-rate.

Implicit constitutive theories certainly allow for a more general structure for material response compared to explicit ones, which, in fact, can be seen as a subclass. Moreover, models with limiting strain provided by implicit constitutive theories are able to capture experimental observations providing theoretical justification for the response of materials such as Gum metal and titanium alloys that were previously proposed in a rather ad hoc manner (see [3] and references therein). Even though some implicit models for describing the elastic response of solids existed for a considerable amount of time, the importance of the cause and the effect in those descriptions was not realized until recently when constitutive models were introduced to describe the response of elastic solids wherein the stress T and the deformation gradient are related implicitly.

In the classical Cauchy elasticity, the stress is a function of the strain B. As a result of linearization of the strain we obtain T=C:ϵ, where : stands for matrix product, C is the fourth order elasticity tensor and ϵ denotes the linearized strain. In conclusion, one could only obtain a linear relation between the stress and the strain, which, however, does not reflect reality in various materials. Here we make use of a framework introduced by Rajagopal [2] in which the strain is considered as a function of the stress, that is, B=f(T). Correspondingly, one considers a nonlinear function for the linearized strain $\epsilon =\tilde{f}(T)$. Hence implicit constitutive theory leads to a small strain theory allowing for a nonlinear relationship between the linearized strain and the stress [4,5].

The notion *strain-limiting* refers to the response of materials which are characterized by the fact that, once the strain reaches a certain limiting value, a further increase in stress will not cause any changes in strain. Such models have recently attracted a considerable amount of attention due to the fact that various phenomena, including cracks, are successfully described. In the classical linear elasticity theory such nonlinear response cannot be explained. The advantage of implicit theories is that they allow for the gradient of the displacement to stay small so that one could treat the linearized strain, even for arbitrary large values of the stress, which has, in fact, been observed in many experiments (see the references in [3]).

In this work, we are interested in the analysis of a one-dimensional viscoelastic model resulting from a constitutive equation specifying the relation between the linearized strain, the stress and the stress-rate. In §2, we introduce the model we study in this work which is derived using the viscoelastic constitutive relation and the equation of motion. This yields an equation for the dynamics of the stress T:Ω×[0,∞)→R with Ω⊂R, cf. (2.5), and reads

1.1 $$h{\left(T\right)}_{tt}-\gamma T_{ttt}=T_{xx}\;\text{on }\Omega \times [0,\infty ).$$

coupled with homogeneous Neumann or periodic boundary conditions for T. Here, γ>0 and the function $h\in C^{2}(R,R)$ is in general nonlinear and satisfies h(0)=0; it reflects the correlation between the strain and the stress of the material.

Duman & Şengül [6] proved the existence of travelling wave solutions for a related case of this partial differential equation (PDE) on R×[0,∞). They considered an equation with the second term in (1.1) being equal to ${\left(\gamma (T)T_{t}\right)}_{tt}$ with γ(⋅) being a function of the stress satisfying certain assumptions. To deal with more general solutions, we consider the case of γ(⋅) being a constant function. We then address the question of classical solutions in the framework of Gevrey classes in which the initial data is not assumed to be a travelling wave.

Next we briefly comment on a related PDE, which is derived for viscoelastic strain-limiting *strain*-rate type models and reads

1.2 $$g{\left(T\right)}_{tt}-\nu T_{xxt}=T_{xx}.$$

The derivation of this equation starts from the equation of motion (2.2) below and uses the constitutive relation $\epsilon +\nu \epsilon_{t}=g(T)$ for some ν>0 and some nonlinear function $g\in C^{2}(R,R)$ satisfying g(0)=0 (see [3,7] for a detailed derivation). For this model, defined on R×[0,∞), travelling wave solutions were studied in [8]. In [9], Şengül found travelling wave solutions analytically and numerically with g(T)=aarctan⁡(bT) for some positive constants a,b∈R. Moreover, local existence of strong solutions was proved for an initial value problem under the assumption that the function g is strictly increasing [10]. Then Şengül [11] proved that these solutions are actually global solutions. The most recent work on (1.2) is by Bachmann *et al.* [12] where a variational framework is introduced for general nonlinearities g. As a result of variational techniques and time-discretization, authors prove the existence of solutions in certain Sobolev spaces when the nonlinearity takes the form g(T)=T.

In §3 of this article, we deal with some well-posedness issues of the equation in the stress-rate case, specifically focusing on the stability of certain steady-state solutions of (1.1) when the domain Ω=T is periodic. To analyse this, we linearize equation (1.1) around constant solutions $T(x,t)=\bar{T}\in R$, leading to

1.3 $$h^{\prime }(\bar{T})T_{tt}-\gamma T_{ttt}=T_{xx}\;\text{on }T\times [0,t_{\max}).$$

Our main result establishes an optimal function space for the existence of local-in-time smooth solutions of (1.3), i.e. up to a maximal time $0<t_{\max}<\infty$. In the variable x∈T, this relates to the so-called Gevrey-class 3/2 regularity. Gevrey classes are function spaces that allow control over an infinite number of derivatives of the functions and have proven to be widely applicable in various contexts, especially in the theory of boundary layers (cf. [13–17]). Our main result asserts the following:

**Theorem 1.1.**

*Assume that the initial data of* T, $T_{t}$ *and* $T_{tt}$ *have regularity Gevrey-class* 3/2 (cf. *definition 3.1 and theorem 3.2*). *Then there exists a lifespan* $t_{\max}>0$ *depending on the initial data and there exists a smooth solution* $T\in C^{\infty }(T\times [0,t_{\max}))$ *of equation* (1.3).

We refer to §3 for details about Gevrey regularities, as well as more insights about the behaviour of the constructed solutions. We emphasize, however, that our analysis determines that the Gevrey-class 3/2 is optimal for equation (1.3) (without any structural assumptions on the initial data). Interestingly, our analysis suggests that the well-posedness in Sobolev spaces is likely to be unattainable for the linearized equation (1.3). This conclusion is drawn from a significant Fourier analysis of the solution frequencies, which highlights that the solutions precisely exhibit a dispersion relation of Gevrey-class 3/2 type. These findings underscore the importance of the Gevrey-class 3/2 regularity in capturing the behaviour of solutions and suggest its optimality for the studied equation. Section 3 provides further elaboration on these insights. Additionally, we refer to §4 for a comprehensive overview on the ill-posedness of (1.3) in Sobolev spaces.

## 2. Introduction to the model

We consider a homogeneous, viscoelastic medium in one space dimension. Let Ω⊂R be a bounded open set. Moreover, let ρ:Ω×[0,∞)→R denote the mass density in space and time, u:Ω×[0,∞)→R the deformation/flow map and T:Ω×[0,∞)→R the (one-dimensional) stress-tensor. The equation of motion then reads

2.1 $$\rho u_{tt}=T_{x}\;\text{on}\;\Omega \times [0,\infty )$$

with homogeneous Dirichlet or periodic boundary conditions for u.

Based on this, we derive an evolution equation for the stress-tensor T=T(x,t) following [7]. By making use of the homogeneity of the material, differentiating (2.1) with respect to the space variable x and assuming suitable regularity of the deformation u, we obtain $\rho u_{xtt}=T_{xx}$. Next we replace $u_{xtt}$ by $\epsilon_{tt}$, where ϵ denotes the (one-dimensional) strain. Hence we obtain

2.2 $$\rho \epsilon_{tt}=T_{xx}\;\text{on}\;\Omega \times [0,\infty ).$$

To write the left-hand side in terms of the stress, we make use of a constitutive equation. We suppose that we have an implicit constitutive equation of the viscoelastic strain-limiting material given by

2.3 $$\epsilon =h(T)-\gamma T_{t}\;\text{on }\Omega \times [0,\infty )$$

for some γ>0 and some nonlinear function $h\in C^{2}(R,R)$ satisfying h(0)=0. Note that we use the shorthand h(T) for h(T(⋅,⋅)). A detailed derivation of this model can be found in [6,7]. Since the constitutive relation also depends on the stress-rate $T_{t}$, the model is also referred to as a stress-rate type model. In [7], Erbay and Şengül showed that this model is consistent with the first and second law of thermodynamics if γ>0. For γ=0, the constitutive relation reduces to the elastic setting. To get an equation of motion only in terms of the stress T and its derivatives, we plug the second time derivative of (2.3) into (2.2) and obtain

2.4 $$\rho {\left(h(T\right)}_{tt}-\gamma T_{ttt})=T_{xx}\;\text{on }\Omega \times [0,\infty )$$

with homogeneous Neumann or periodic boundary conditions for T. Using the dimensionless quantities introduced in [7], we finally get the PDE

2.5 $$h{\left(T\right)}_{tt}-\gamma T_{ttt}=T_{xx}\;\text{on }\Omega \times [0,\infty ).$$

Note that, by an abuse of standard notation, the time derivatives of h(T) are total derivatives. However, for ϵ and T there is no difference between total and partial derivatives since the spatial variable x is independent of t.

## 3. Gevrey-type solutions of the linearized model

In this section, we focus on addressing the issue of well-posedness associated with a proper linearization of equation (2.5). We here consider a simplified geometry, where the domain Ω=T=[−π,π] represents a one-dimensional periodic torus. This choice allows us to circumvent certain complications arising from boundary conditions. However, even in this simplified geometry, we provide evidence of the presence of certain instability mechanisms in the solutions.

We linearize (2.5) around meaningful steady-state solutions, which in this case are represented by constant functions $T(x,t)=\bar{T}\in R$, for any (x,t)∈T×[0,∞). An important question arises as to whether these constant solutions remain stable under small perturbations. In other words, we investigate whether the following linearized equation is well-posed, at least within a local time frame:

3.1 $$h^{\prime }(\bar{T})T_{tt}-\gamma T_{ttt}=T_{xx}\;(x,t)\in T\times [0,t_{\max}).$$

The analysis of (3.1) can indeed offer valuable insights on its nonlinear counterpart (2.5). For now, let us consider the lifespan $t_{\max}>0$ as an arbitrary positive time. However, we determine an explicit formula for $t_{\max}$ based on appropriate initial data (as indicated in (3.4)).

We demonstrate that certain inherent instability mechanisms complicate and limit the function settings for which (3.1) has a local-in-time solution. This requires the control of an infinite number of derivatives of the initial data.

Due to the periodic nature of T, we can employ the Fourier series decomposition to represent a suitable solution T=T(x,t) (including also its initial data):

$$T(x,t)=\sum_{k\in Z}T_{k}(t)e^{ikx},\;T(x,0)=T_{\mathrm{in}}(x)=\sum_{k\in Z}T_{\mathrm{in},k}e^{ikx},$$

$$T_{t}(x,0)=T_{t,\mathrm{in}}(x)=\sum_{k\in Z}T_{t,\mathrm{in},k}e^{ikx},\;T_{tt}(x,0)=T_{tt,\mathrm{in}}(x)=\sum_{k\in Z}T_{tt,\mathrm{in},k}e^{ikx}.$$

For a general function $f\in L^{1}(T)$ with $f(x)=\sum_{k\in Z}f_{k}e^{ikx},x\in T$ (integrability is a minimal assumption to define at least a weak solutions), the Fourier coefficients satisfy $f_{k}:=(1/2\pi )\int_{T}f(x)e^{-ikx}\;dx$. (Certainly the Fourier series is well defined also for more general distributions, but this is beyond the interest of this paper.)

A common approach to control an infinite amount of derivatives of the initial data $T_{\mathrm{in}}$, $T_{t,\mathrm{in}}$, $T_{tt,\mathrm{in}}$ is to impose a suitable decay on the corresponding Fourier coefficients, as the frequencies k→±∞. When these coefficients decay exponentially as $e^{-\sigma |k|}$, for a fixed σ>0, the functions involved are analytic and can be locally represented as power series. However, we are interested in more refined initial data, that exhibit regularities between analytic and Sobolev spaces.

Specifically, we consider initial data with Gevrey-class 3/2 regularity. Gevrey spaces have recently attracted attention in the mathematical community, particularly in relation to well-posedness issues in models involving boundary layers (cf. [13–19]). Roughly speaking, the initial data belongs to the Gevrey-class 3/2 if the corresponding Fourier coefficients decay as $e^{-\sigma |k{|}^{2/3}}$, when the frequencies diverge. More generally, we have the following definition :

**Definition 3.1.**

A function $f\in L^{1}(T)$ belongs to the Gevrey-class $G_{\sigma }^{m}=G_{\sigma }^{m}(T)$, for fixed σ>0 and m>1, if the following norm is bounded:

$$||f|{|}_{G_{\sigma }^{m}}:=\underset{k\in Z}{\sup}\{e^{\sigma |k{|}^{1/m}}|f_{k}|\}<\infty ,\;\text{where}\;f(x)=\sum_{k\in Z}f_{k}e^{ikx}\;\text{with}\;f_{k}=\frac{1}{2\pi }\int_{T}f(x)e^{-ikx}\;dx.$$

For comprehensive explanations on Gevrey classes by means of Fourier Analysis, we refer to Rodino [20].

Before stating our main result, some remarks are here in order. Because of the exponential decay on the modes, functions in the class $G_{\sigma }^{m}$ are also $C^{\infty }(T)$. We aim therefore to generate smooth solutions of (3.1) in $C^{\infty }(T)\times [0,t_{\max})$, for a suitable lifespan $t_{\max}>0$ depending upon the initial data. The exponent m=3/2 is critical and was already observed by the last author in [7] (cf. Section 5.3).

Given that γ>0, we introduce a formal time rescaling of the stress variable T(x,t) using $\tilde{T}(x,t):=T(x,\gamma^{1/3}t)$ and define

$$\alpha :=h^{\prime }(\bar{T})\gamma^{-(2/3)}.$$

Additionally, we rescale the lifespan by means of ${\tilde{t}}_{\text{max}}:=\gamma^{-(1/3)}t_{\text{max}}$ and, with an abuse of notation, we omit the symbols ∼ denoting the rescaled quantities. Consequently, the resulting PDE is as follows:

3.2 $$\left\{ \begin{array}{rl} & \alpha T_{tt}-T_{ttt}=T_{xx},\;(x,t)\in T\times [0,t_{\max}),\\ & T{|}_{t=0}=T_{\mathrm{in}},\;\;\;x\in T,\\ & T_{t}{|}_{t=0}=T_{t,\mathrm{in}},\;\;\;x\in T,\\ & T_{tt}{|}_{t=0}=T_{tt,\mathrm{in}},\;x\in T. \end{array}\right.$$

**Theorem 3.2.**

*Assume that* $T_{\text{in}}$, $T_{t,\mathrm{in}}$ *and* $T_{tt,\mathrm{in}}$ *belong to the Gevrey-class* $G_{\sigma }^{3/2}$, *for a suitable radius* σ>0, *as described by definition 3.1. Then there exists a local-in-time smooth solution* T=T(x,t) *of* (3.2), *such that*

3.3 $$T\in L^{\infty }(0,t_{\max};G_{\beta (t)}^{3/2}),\;\partial_{t}^{m}T\in L^{\infty }(0,t_{\max};G_{\Gamma (t)}^{3/2})\;\text{for any }m\in N,$$

*where the lifespan* $t_{\text{max}}$ *and the radii of Gevrey regularity* β(t),Γ(t) *are defined by*

3.4 $$t_{\max}:=2^{-(4/3)}\sigma ,\;\beta (t):=\sigma -2^{1/3}t>0,\;\Gamma (t):=\frac{\sigma }{2}-2^{1/3}t>0\;\text{for any}\;t\in [0,t_{\max}).$$

**Remark 3.3.**

Before addressing the proof of theorem 3.2, some remarks about the statement are here in order. The radii β, Γ of Gevrey regularity in (3.4) are decreasing functions in time, which indicates a specific ‘loss of regularity’ as time progresses. This reflects a mechanism of instability inherent in the solutions. In essence, without structural assumptions on the initial data, we will determine a dispersion relation on the frequencies of order $k^{2/3}$ (cf. $\lambda_{1,k}$ *in remark 3.7, as well as lemma 3.4), that can only be counteracted (locally in time) by initial data belonging to the Gevrey-class* 3/2 *or higher. Discussions regarding lower regularities and insights into the ill-posedness in Sobolev spaces will be addressed in §4*.

The function spaces in (3.3) define a function T=T(x,t), which is indeed smooth both in time and space (thus T is a classical solution). For any radii of regularity 0<η<σ and any s>1, the following Gevrey embedding holds true: $G_{\sigma }^{s}↪G_{\eta }^{s}↪C^{\infty }(T)$. If we set $0<t^{\prime }<t_{\text{max}}$ and $\eta :=\Gamma (t^{\prime })$, we gather that

$$G_{\beta (t)}^{3/2}↪G_{\Gamma (t)}^{3/2}↪G_{\eta }^{3/2}\;\text{for any }t\in [0,t^{\prime }].$$

This embedding together with (3.3) implies in particular that T belongs to $W^{m,\infty }(0,t^{\prime };G_{\eta }^{3/2})$, for any m∈N, and therefore also to $C^{\infty }(T\times [0,t^{\prime }])$. The arbitrariness of $t^{\prime }\in (0,t_{\text{max}})$ leads therefore to $T\in C^{\infty }(T\times [0,t_{\text{max}}))$, hence T is a smooth solution of (3.2).

**Proof.**

We begin with by projecting the main equation (3.2) to the eigenspace generated by the oscillating function ${\text{e}}^{\text{i}kx}$ for some k fixed. For each frequency k∈Z, the Fourier coefficient $T_{k}(t)$ depends only on time and is *a priori* a solution of the following ordinary differential equation:

3.5 $$\left\{ \begin{array}{rl} & T_{k}^{\prime \prime \prime }(t)-\alpha T_{k}^{\prime \prime }(t)-k^{2}T_{k}(t)=0,\;t\in R,\\ & T_{k}{|}_{t=0}=T_{\mathrm{in},k}\in R,\\ & T_{k,t}{|}_{t=0}=T_{t,\mathrm{in},k}\in R,\\ & T_{k,tt}{|}_{t=0}=T_{tt,\mathrm{in},k}\in R. \end{array}\right.$$

For a fixed k∈Z, equation (3.5) is linear, and of order three. It admits therefore a global-in-time solution $T_{k}\in C^{\infty }(R)$, which can be determined by making use of the characteristic equation (3.7). The lifespan $t_{\text{max}}>0$ will be discussed later in the context of ensuring the convergence of the Fourier series in a specific manner. The solution $T_{k}(t)$ is explicitly determined by

3.6 $$T_{k}(t)=c_{1,k}\;e^{\lambda_{1,k}t}+c_{2,k}\;e^{\lambda_{2,k}t}+c_{3,k}\;e^{\lambda_{3,k}t},$$

where $\lambda =\lambda_{1,k},\lambda_{2,k},\lambda_{3,k}$ are the three distinct complex roots of the following characteristic polynomial of degree three:

3.7 $$p(\lambda ):=\lambda^{3}-\alpha \lambda^{2}-k^{2}=0.$$

While it is indeed possible to explicitly determine the values of $\lambda =\lambda_{1,k},\lambda_{2,k},\lambda_{3,k}$ (cf. remark 3.7), our primary interest lies in understanding their behaviour as the frequencies become increasingly higher. This aspect provides valuable insights into the convergence properties of the Fourier series and, consequently, the function spaces in which a solution exists. By examining the behaviour of λ with respect to high frequencies, we can gain a deeper understanding of the convergence behaviour and the appropriate function spaces for the solution. The constants $c_{1,k}$, $c_{2,k}$ and $c_{3,k}$ in (3.6) depend uniquely upon the initial data $T_{\text{in, k}}$, $T_{t,\mathrm{in},k}$, $T_{tt,\mathrm{in},k}$ and satisfy

$$\begin{array}{rl} & c_{1,k}+c_{2,k}+c_{3,k}=T_{\mathrm{in},\;k},\\ & \lambda_{1,k}c_{1,k}+\lambda_{2,k}c_{2,k}+\lambda_{3,k}c_{3,k}=T_{t,\mathrm{in},k}\\ \mathrm{and}\; & \lambda_{1,k}^{2}c_{1,k}+\lambda_{2,k}^{2}c_{2,k}+\lambda_{3,k}^{2}c_{3,k}=T_{tt,\mathrm{in},k}. \end{array}$$

Consequently, we can derive the following expression:

3.8 $$\begin{array}{rl} & c_{1,k}=\frac{T_{tt,\mathrm{in},k}-(\lambda_{3,k}+\lambda_{2,k})T_{t,\mathrm{in},k}+\lambda_{2,k}\lambda_{3,k}T_{\mathrm{in},k}}{\left(\lambda_{1,k}-\lambda_{2,k})(\lambda_{1,k}-\lambda_{3,k}\right)},\\ & c_{2,k}=\frac{T_{tt,\mathrm{in},k}-(\lambda_{3,k}+\lambda_{1,k})T_{t,\mathrm{in},k}+\lambda_{1,k}\lambda_{3,k}T_{\mathrm{in},k}}{\left(\lambda_{2,k}-\lambda_{3,k})(\lambda_{2,k}-\lambda_{1,k}\right)},\\ & c_{3,k}=-\frac{T_{tt,\mathrm{in},k}-(\lambda_{2,k}+\lambda_{1,k})T_{t,\mathrm{in},k}+\lambda_{1,k}\lambda_{2,k}T_{\mathrm{in},k}}{\left(\lambda_{1,k}-\lambda_{3,k})(\lambda_{3,k}-\lambda_{2,k}\right)}. \end{array}$$

Note here that $\lambda_{i,k}\neq \lambda_{j,k}$ holds whenever i≠j (we will guarantee this fact later on, with the result of lemma 3.4), hence the above expressions are well defined. The roots $\lambda_{1,k},\lambda_{2,k}$ and $\lambda_{3,k}$ are indeed distinct for each value of the frequency k.

To estimate the Fourier coefficient $T_{k}(t)$ in terms of the frequencies k∈Z, we need to establish some related estimates for the roots $\lambda_{1,k},\lambda_{2,k}$ and $\lambda_{3,k}$. This will allow us to gain insights into their behaviour as the frequencies increase. We assert in particular that the following estimates hold true: for any k∈Z with $|k|>2{\left(1+|\alpha |\right)}^{3/2}$

3.9 $$2^{-(1/3)}|k{|}^{2/3}\leq \lambda_{1,k}\leq 2^{1/3}|k{|}^{2/3},\;|\lambda_{2,k}|=|\lambda_{3,k}|\leq 2|k{|}^{2/3}.$$

For clarity of the proof of theorem 3.2, we postpone the proof of these estimates to lemma 3.4 and remark 3.5 below.

By (3.9) we obtain that the first term on the r.h.s. in (3.6) satisfies

3.10 $$|c_{1,k}e^{\lambda_{1,k}t}|\leq |c_{1,k}|e^{2^{1/3}|k{|}^{2/3}t},$$

for any k∈Z, with $|k|>2{\left(1+|\alpha |\right)}^{3/2}$. Similarly, the second and third terms on the r.h.s. in (3.6) fulfil

3.11 $$\begin{array}{rl} & \;|c_{2,k}e^{\lambda_{2,k}t}|=|c_{2,k}|e^{ℜ(\lambda_{2,k})t}=|c_{2,k}|e^{(-(\lambda_{1,k}-\alpha )/2)t}\leq |c_{2,k}|e^{-2^{-(4/3)}|k{|}^{2/3}t+\frac{\alpha }{2}t},\\ & \;|c_{3,k}e^{\lambda_{3,k}t}|=|c_{3,k}|e^{ℜ(\lambda_{3,k})t}=|c_{3,k}|e^{(-(\lambda_{1,k}-\alpha )/2)t}\leq |c_{3,k}|e^{-2^{-(4/3)}|k{|}^{2/3}t+(\alpha /2)t}, \end{array}$$

for any k∈Z, with $|k|>2{\left(1+|\alpha |\right)}^{3/2}$. In particular, we obtain that each mode $T_{k}(t)$ satisfies

3.12 $$|T_{k}(t)|\leq 3\max\{|c_{1,k}|,\;|c_{2,k}|,\;|c_{3,k}|\}e^{2^{1/3}|k{|}^{2/3}t},$$

at any time t>0. The obtained estimates (3.10), (3.11) and (3.12) already contain significant information regarding the potential regularity of the solution T=T(x,t). Notably, the root $\lambda_{1,k}$ serves as a primary indicator of the solution’s instability. It leads to an exponential upper bound in the modes of order $|k{|}^{2/3}t$ in the frequencies k≫1 (which can be counteracted locally in time by a regularity Gevrey 3/2 on the initial data, at best).

Contrarily, the second and third roots $\lambda_{2,k}$ and $\lambda_{3,k}$ behave roughly as $∼{\text{e}}^{-|k{|}^{2/3}t}$ at high frequencies, which further supports the decay of the modes of the initial data (hence producing a sort of smoothing effect, cf. also §4). To explore this expected behaviour in more detail, we establish a relationship between the growth of the constants $c_{1,k},c_{2,k},c_{3,k}\in C$ and the modes $T_{\text{in},k},T_{t,\text{in},k},T_{tt,\text{in},k}$. Once again, our focus will be on understanding the behaviour of these constants at high frequencies. We assert that for any k∈Z with $|k|>2{\left(1+|\alpha |\right)}^{3/2}$ there exists a constant C>0 such that

3.13 $$\max\{|c_{1,k}|,\;|c_{2,k}|,\;|c_{3,k}|\}\leq C\max\{||T_{\mathrm{in}}|{|}_{G_{\sigma }^{3/2}},||T_{t,\mathrm{in}}|{|}_{G_{\sigma }^{3/2}},||T_{tt,\mathrm{in}}|{|}_{G_{\sigma }^{3/2}}\}e^{-\sigma |k{|}^{2/3}}.$$

The proof of this estimate is deferred to lemma 3.6, and we now shift our attention to concluding the proof of theorem 3.2. From now on, we allow the constant C to eventually change from line to line.

Invoking the definition of the lifespan $t_{\text{max}}=2^{-(4/3)}\sigma >0$ and the radius $\beta (t)=\sigma -2^{1/3}t$ in (3.4), as well as the coefficients $T_{k}(t)$ in (3.6), we assert first that the Fourier series

$$T(t,x):=\sum_{k\in Z}T_{k}(t)e^{ikx}=\sum_{|k|\leq 2{\left(1+|\alpha |\right)}^{3/2}}T_{k}(t)e^{ikx}+\sum_{|k|>2{\left(1+|\alpha |\right)}^{3/2}}T_{k}(t)e^{ikx},$$

converges in the space $G_{\beta (t)}^{3/2}$ for any $t\in [0,t_{\text{max}})$. To this end, we split the Gevrey norm into the contribution of low and high frequencies:

$$\underset{k\in Z}{\sup}\{e^{\beta (t)|k{|}^{2/3}}|T_{k}(t)|\}\leq \underset{|k|\leq 2{\left(1+|\alpha |\right)}^{3/2}}{\sup}\{e^{\beta (t)|k{|}^{2/3}}|T_{k}(t)|\}+\underset{|k|>2{\left(1+|\alpha |\right)}^{3/2}}{\sup}\{e^{\beta (t)|k{|}^{2/3}}|T_{k}(t)|\}.$$

The first supremum is between a finite amount of frequencies (hence it is finite, being indeed a maximum), while the second supremum determines whether the Gevrey-3/2 norm is bounded or not. By invoking (3.12), we gather that

3.14 $$\begin{array}{rl} \underset{|k|>2{\left(1+|\alpha |\right)}^{3/2}}{\sup} & \;\{e^{\beta (t)|k{|}^{2/3}}|T_{k}(t)|\}\\ & \;\;\leq \underset{|k|>2{\left(1+|\alpha |\right)}^{3/2}}{\sup}\{e^{\beta (t)|k{|}^{2/3}+2^{1/3}|k{|}^{2/3}t}3\max\{|c_{1,k}|,|c_{2,k}|,|c_{3,k}|\}\}. \end{array}$$

Hence, recalling from (3.4) that $\beta (t)=\sigma -2^{1/3}t$ and thanks to the estimate in (3.13), we obtain that

$$\begin{array}{rl} \underset{|k|>2{\left(1+|\alpha |\right)}^{3/2}}{\sup} & \;\{e^{\beta (t)|k{|}^{2/3}}|T_{k}(t)|\}\\ & \;\;\leq \underset{|k|>2{\left(1+|\alpha |\right)}^{3/2}}{\sup}\{e^{\sigma |k{|}^{2/3}}C\max\{||T_{\mathrm{in}}|{|}_{G_{\sigma }^{3/2}},||T_{t,\mathrm{in}}|{|}_{G_{\sigma }^{3/2}},||T_{tt,\mathrm{in}}|{|}_{G_{\sigma }^{3/2}}\}e^{-\sigma |k{|}^{2/3}}\}\\ & \;\;\leq C\max\{||T_{\mathrm{in}}|{|}_{G_{\sigma }^{3/2}},||T_{t,\mathrm{in}}|{|}_{G_{\sigma }^{3/2}},||T_{tt,\mathrm{in}}|{|}_{G_{\sigma }^{3/2}}\}<+\infty . \end{array}$$

This finally implies that $T(x,t)=\sum_{k\in Z}T_{k}(t){\text{e}}^{\text{i}kx}$ converges in $L^{\infty }(0,t_{\text{max}};G_{\beta (t)}^{3/2})$.

We infer also that all time derivatives $\partial_{t}^{m}T$, with m∈N, converge in $L^{\infty }(0,t_{\text{max}};G_{\Gamma (t)}^{3/2})$, where Γ(t)=β(t)−σ/2<β(t). In essence, by decreasing the Gevrey radius of regularity from β(t) to Γ(t) (while ensuring with the lifespan $t_{\text{max}}$ that both radii are positive), we can localize an exponential decay ${\text{e}}^{-\sigma |k{|}^{2/3}/2}$ that counteracts any polynomial growth in the frequencies that arises during the derivation of the Fourier series in time. When taking the derivative with respect to time, we essentially multiply each component of the modes $T_{k}(t)$ with the roots $\lambda_{1,k}$, $\lambda_{2,k}$ and $\lambda_{3,k}$, which grow at most polynomially as |k|≫1. (It is worth noting that higher derivatives $\partial_{t}^{m}T$ also involve multiplications with $\lambda_{1,k}^{m}$, $\lambda_{2,k}^{m}$ and $\lambda_{3,k}^{m}$, which still exhibit polynomial growth.)

To estimate $\partial_{t}T$, we decompose once more the associated Fourier series into high and low frequencies:

3.15 $$\sum_{k\in Z}T_{k}^{\prime }(t)e^{ikx}=\sum_{|k|\leq 2{\left(1+|\alpha |\right)}^{3/2}}T_{k}^{\prime }(t)e^{ikx}+\sum_{|k|>2{\left(1+|\alpha |\right)}^{3/2}}T_{k}^{\prime }(t)e^{ikx}$$

and we show that the series converges in the Gevrey class $G_{\Gamma (t)}^{3/2}$, for any time $t\in [0,t_{\text{max}})$. The low frequencies in (3.15) generate a finite sum, which is indeed an analytic function (thus also in any Gevrey class). We need therefore to address the high frequencies k∈Z, with $|k|>2{\left(1+|\alpha |\right)}^{3/2}$.

We proceed with a similar argument as the one used to tackle the function T=T(x,t) and we show that the Gevrey norm of the Fourier series defined by $\partial_{t}T$ is indeed bounded. First, from the definition of $T_{k}(t)$ in (3.6), we obtain that

$$T_{k}^{\prime }(t)=c_{1,k}\lambda_{1,k}e^{\lambda_{1,k}t}+c_{2,k}\lambda_{2,k}e^{\lambda_{2,k}t}+c_{3,k}\lambda_{3,k}e^{\lambda_{3,k}t},$$

which implies in particular that

$$|T_{k}^{\prime }(t)|\leq 3\max\{|c_{1,k}|,|c_{2,k}|,|c_{3,k}|\}\max\{|\lambda_{1,k}|,|\lambda_{2,k}|,|\lambda_{3,k}|\}e^{2^{1/3}|k{|}^{2/3}t}.$$

The exponential growth depends on time and is derived similarly as in (3.12) from the terms ${\text{e}}^{\lambda_{j,k}t}$, j=1,2,3. We invoke hence (3.13) together with the estimates in (3.9) to obtain that

$$|T_{k}^{\prime }(t)|\leq C\max\{||T_{\mathrm{in}}|{|}_{G_{\sigma }^{3/2}},||T_{t,\mathrm{in}}|{|}_{G_{\sigma }^{3/2}},||T_{tt,\mathrm{in}}|{|}_{G_{\sigma }^{3/2}}\}e^{-\sigma |k{|}^{2/3}}2|k{|}^{2/3}e^{2^{1/3}|k{|}^{2/3}t}.$$

Remark that contrarily to (3.12), we here have some polynomial-type growth in the frequencies that has to be controlled by the exponential decay (hence the need for a lower radius of Gevrey regularity). Recalling that $\Gamma (t)=(\sigma /2)-2^{1/3}t$ we have that

$$\begin{array}{rl} & \;\underset{|k|>2{\left(1+|\alpha |\right)}^{3/2}}{\sup}\{e^{\Gamma (t)|k{|}^{2/3}}|T_{k}^{\prime }(t)|\}\\ & \;\;\leq C\underset{|k|>2{\left(1+|\alpha |\right)}^{3/2}}{\sup}\{e^{((\sigma /2)-2^{1/3}t)|k{|}^{2/3}}\max\{||T_{\mathrm{in}}|{|}_{G_{\sigma }^{3/2}},||T_{t,\mathrm{in}}|{|}_{G_{\sigma }^{3/2}},||T_{tt,\mathrm{in}}|{|}_{G_{\sigma }^{3/2}}\}e^{(-\sigma +2^{1/3})|k{|}^{2/3}}|k{|}^{2/3}\}\\ & \;\;\leq C\underset{|k|>2{\left(1+|\alpha |\right)}^{3/2}}{\sup}\{|k{|}^{2/3}e^{-(\sigma /2)|k{|}^{2/3}}\}\max\{||T_{\mathrm{in}}|{|}_{G_{\sigma }^{3/2}},||T_{t,\mathrm{in}}|{|}_{G_{\sigma }^{3/2}},||T_{tt,\mathrm{in}}|{|}_{G_{\sigma }^{3/2}}\}. \end{array}$$

Finally, we remark that $a{\text{e}}^{-a}\leq 1$ holds for any real number a∈R, hence setting $a=\sigma /2|k{|}^{2/3}$, we gather

$$\underset{|k|>2{\left(1+|\alpha |\right)}^{3/2}}{\sup}\{e^{\Gamma (t)|k{|}^{2/3}}|T_{k}^{\prime }(t)|\}\leq \frac{C}{\sigma }\max\{||T_{\mathrm{in}}|{|}_{G_{\sigma }^{3/2}},||T_{t,\mathrm{in}}|{|}_{G_{\sigma }^{3/2}},||T_{tt,\mathrm{in}}|{|}_{G_{\sigma }^{3/2}}\}.$$

Since this last term is bounded, the series of $\partial_{t}T$ defined in (3.15) converges indeed in $L^{\infty }(0,t_{\text{max}};G_{\Gamma (t)}^{3/2})$.

We infer that a similar argument implies also that the series generated by $\partial_{t}^{m}T$, with m∈N, converge always in $L^{\infty }(0,t_{\text{max}};G_{\Gamma (t)}^{3/2})$. This in particular implies that the function T belongs also to $C^{\infty }(T\times [0,t_{\text{max}}))$, hence it is smooth.

In conclusion, we note that the constructed function T=T(x,t) in the function spaces specified in (3.3) is indeed a weak solution of the main system (3.2), as it satisfies the equation in each eigenspace generated by ${\text{e}}^{\text{i}kx}$. Moreover, since the function T is smooth, we obtain that T is, in fact, a smooth solution of (3.2). This completes the proof of theorem 3.2.

To conclude this section, we shall now devote our attention to the proof of the estimates in (3.9) and (3.13). Throughout the forthcoming estimates, we will repeatedly consider high frequencies satisfying $|k|>2{\left(1+|\alpha |\right)}^{3/2}$. We begin with the following lemma for (3.9).

**Lemma 3.4.**

*For every* k∈Z *satisfying* $|k|>2{\left(1+|\alpha |\right)}^{3/2}$, *the three distinct roots given by* $\lambda =\lambda_{1,k},\;\lambda_{2,k},\;\lambda_{3,k}\in C$ *of the polynomial* $p(\lambda ):=\lambda^{3}-\alpha \lambda^{2}-k^{2}$ *satisfy the following properties (in case, commuting the order of the roots*):

(i) *The first root* $\lambda_{1,k}$ *is a real positive number, which increases as* $|k{|}^{2/3}$, *as the frequencies diverge. In particular*, $\lambda_{1,k}$ *is uniformly bounded from above and below through the following inequalities*:
  3.16 $$2^{-\frac{1}{3}}|k{|}^{2/3}\leq \lambda_{1,k}\leq 2^{1/3}|k{|}^{2/3}.$$
(ii) *The complex roots* $\lambda_{2,k}$ *and* $\lambda_{3,k}$ *have non-trivial imaginary parts and they are conjugated. They can be explicitly written in terms of the first root* $\lambda_{1,k}$ *by means of*
  3.17 $$\begin{array}{rl} & \lambda_{2,k}=-\frac{\lambda_{1,k}-\alpha }{2}+\frac{i}{2}\sqrt{\left(\lambda_{1,k}-\alpha )(3\lambda_{1,k}+\alpha \right)},\\ & \lambda_{3,k}=-\frac{\lambda_{1,k}-\alpha }{2}-\frac{i}{2}\sqrt{\left(\lambda_{1,k}-\alpha )(3\lambda_{1,k}+\alpha \right)}. \end{array}$$

**Proof.**

We aim to determine an appropriate behaviour of the roots of the polynomial

$$p(\lambda )=\lambda^{3}-\alpha \lambda^{2}-k^{2}.$$

Since p has a degree of 3, it will have three complex roots, and at least one of them will be real. Indeed, since p has real coefficients, for any complex root λ, its conjugate $\bar{\lambda }$ will also be a root of p.

To determine the behaviour of the real root of p (which we denote $\lambda =\lambda_{1,k}\in R$) we make use of the intermediate value theorem, stating that if a continuous function changes sign between two values, then it must have a root in between those values. To apply the intermediate value theorem, we consider three separate cases: when α=0, when α>0 and when α<0.

In the case of α=0, the analysis is straightforward. The real root $\lambda_{1,k}$ coincides with $|k{|}^{2/3}$, which indeed satisfies the estimate (3.16).

For α>0, we set two distinct values $\lambda =|k{|}^{2/3}$ and $\lambda =2^{1/3}|k{|}^{2/3}$. Recalling that $|k|>2{\left(1+|\alpha |\right)}^{3/2}$, we get that $|k{|}^{2/3}>2^{2/3}(1+|\alpha |)$ and furthermore

$$p(|k{|}^{2/3})={\left(|k{|}^{2/3}\right)}^{3}-\alpha {\left(|k{|}^{2/3}\right)}^{2}-k^{2}=-\alpha |k{|}^{4/3}<0$$

and

$$p(2^{1/3}|k{|}^{2/3})={\left(2^{1/3}|k{|}^{2/3}\right)}^{3}-\alpha {\left(2^{1/3}|k{|}^{2/3}\right)}^{2}-k^{2}=|k{|}^{4/3}(|k{|}^{2/3}-2^{2/3}\alpha )>0.$$

From this we can conclude that there is a real root of the polynomial in the interval $\left[|k{|}^{2/3},2^{1/3}|k{|}^{2/3}\right]$ (thus also in $\left[2^{-(1/3)}|k{|}^{2/3},2^{1/3}|k{|}^{2/3}\right]$), thanks to the intermediate value theorem.

Next we address the case of a negative α<0, always under the restriction $|k|>2{\left(1+|\alpha |\right)}^{3/2}$. We first consider $\lambda =|k{|}^{2/3}$ and then $\lambda =2^{-(1/3)}|k{|}^{2/3}$:

$$p(|k{|}^{2/3})=-\alpha |k{|}^{4/3}>0$$

and

$$p(2^{-(1/3)}|k{|}^{2/3})={\left(2^{-(1/3)}|k{|}^{2/3}\right)}^{3}-\alpha {\left(2^{-(1/3)}|k{|}^{2/3}\right)}^{2}-k^{2}=-\frac{1}{2}|k{|}^{4/3}(|k{|}^{2/3}+2^{1/3}\alpha )<0.$$

From this we can conclude that there is a real root of the polynomial in the interval $\left[2^{-(1/3)}|k{|}^{2/3},|k{|}^{2/3}\right]$ (thus also in $\left[2^{-(1/3)}|k{|}^{2/3},2^{1/3}|k{|}^{2/3}\right]$).

Summarising, it follows that for every α∈R and for $|k|>2{\left(1+\alpha \right)}^{3/2}$ the polynomial has a real root satisfying

$$2^{-(1/3)}|k{|}^{2/3}\leq \lambda_{1,k}\leq 2^{1/3}|k{|}^{2/3},$$

which proves statement (i).

We next address the statement (ii) in lemma 3.4. We recast the polynomial p by means of the following factorization:

$$p(\lambda )=(\lambda -\lambda_{1,k})(\lambda^{2}+(\lambda_{1,k}-\alpha )\lambda +(\lambda_{1,k}-\alpha )\lambda_{1,k}).$$

To determine the complex values of $\lambda_{2,k}$ and $\lambda_{3,k}$, we look for the roots of the quadratic polynomial $\lambda^{2}+(\lambda_{1,k}-\alpha )\lambda +(\lambda_{1,k}-\alpha )\lambda_{1,k}$. These roots can be expressed explicitly in terms of the real root $\lambda_{1,k}$ as follows:

$$\lambda_{2/3,k}=-\frac{\lambda_{1,k}-\alpha }{2}\pm \frac{\sqrt{\lambda_{1,k}-\alpha }}{2}\sqrt{-3\lambda_{1,k}-\alpha }.$$

We can hence rewrite $\lambda_{2/3,k}$ as

$$\lambda_{2/3,k}=-\frac{\lambda_{1,k}-\alpha }{2}\pm \frac{i}{2}\sqrt{\lambda_{1,k}-\alpha }\sqrt{3\lambda_{1,k}+\alpha }.$$

Furthermore, since $|k|>2{\left(1+|\alpha |\right)}^{3/2}$, both $\lambda_{1,k}-\alpha >0$ and $3\lambda_{1,k}+\alpha >0$ (cf. remark (3.5)), hence the imaginary parts of $\lambda_{2,k}$ and $\lambda_{3,k}$ are not trivial. This concludes the proof of the lemma.

**Remark 3.5.**

From lemma 3.4 we may further derive an upper bound for $|\lambda_{2,k}|$, $|\lambda_{3,k}|$. Thanks to (3.16) and by considering frequencies k∈Z with $|k|>2{\left(1+|\alpha |\right)}^{3/2}$, we remark that the expressions in (3.17) of $\lambda_{2,k}$ and $\lambda_{3,k}$ have non-trivial imaginary parts, since $\lambda_{1,k}\in R$ from lemma 3.4 and also

$$\lambda_{1,k}-\alpha \geq 2^{-(1/3)}|k{|}^{2/3}-\alpha \geq 2^{-(1/3)}2^{2/3}(1+|\alpha |)-\alpha \geq 1>0$$

and

$$3\lambda_{1,k}+\alpha \geq 3\cdot 2^{-(1/3)}|k{|}^{2/3}+\alpha \geq 3\cdot 2^{1/3}(1+|\alpha |)+\alpha \geq 3>0,$$

implying that $-(1/2)(\lambda_{1,k}-\alpha )\in R$ and $\left(1/2)\sqrt{\left(\lambda_{1,k}-\alpha )(3\lambda_{1,k}+\alpha \right)}\in R\setminus \{0\right\}$. Hence, a direct calculation together with the upper bound of $|\lambda_{1,k}|$ in (3.16) imply that

$$\begin{array}{rl} |\lambda_{2,k}{|}^{2} & \;=|\lambda_{3,k}{|}^{2}=\frac{{\left(\lambda_{1,k}-\alpha \right)}^{2}}{4}+\frac{1}{4}(\lambda_{1,k}-\alpha )(3\lambda_{1,k}+\alpha )\\ & \;=\lambda_{1,k}(\lambda_{1,k}-\alpha )\leq 2^{1/3}|k{|}^{2/3}(2^{1/3}|k{|}^{2/3}+2^{-(1/3)}|k{|}^{2/3})=(1+2^{2/3})|k{|}^{4/3}\leq 4|k{|}^{4/3}. \end{array}$$

This yields in particular

3.18 $$|\lambda_{2,k}|=|\lambda_{3,k}|\leq 2|k{|}^{2/3}.$$

To conclude this section, we now provide the proof of the estimate in (3.13), which is the last necessary result to support the statements made in theorem 3.2. We proceed with the following lemma.

**Lemma 3.6.**

*There exists a constant* C>0 *such that the coefficients* $c_{1,k}$, $c_{2,k}$, $c_{3,k}$ *defined in* (3.8) *satisfy*

$$\max\{|c_{1,k}|,\;|c_{2,k}|,\;|c_{3,k}|\}\leq C\max\{||T_{\mathrm{in}}|{|}_{G_{\sigma }^{3/2}},||T_{t,\mathrm{in}}|{|}_{G_{\sigma }^{3/2}},||T_{tt,\mathrm{in}}|{|}_{G_{\sigma }^{3/2}}\}e^{-\sigma |k{|}^{2/3}},$$

*for any frequencies* k∈Z *satisfying* $|k|>2{\left(1+|\alpha |\right)}^{3/2}$ (*hence also at high frequencies* |k|≫1).

**Proof.**

We aim to find suitable bounds for the constants $c_{1,k},c_{2,k},c_{3,k}$ given in (3.8) depending on the roots of the polynomial of the characteristic problem and the initial data. To this end we will frequently use the bounds for $\lambda_{1,k}$ from lemma 3.4, the bound for $\lambda_{2,k},\lambda_{3,k}$ from remark 3.5 and the assumption

3.19 $$|k|>2{\left(1+|\alpha |\right)}^{3/2}>2|\alpha {|}^{3/2}.$$

We begin with by estimating the denominators of the constants $c_{1,k}$, $c_{2,k}$, $c_{3,k}$. We get

3.20 $$\begin{array}{rl} |\lambda_{1,k}-\lambda_{2,k}{|}^{2} & \;=\frac{9}{4}\lambda_{1,k}^{2}-\frac{3}{2}\lambda_{1,k}\alpha +\frac{\alpha^{2}}{4}+\frac{1}{4}(\lambda_{1,k}-\alpha )(3\lambda_{1,k}+\alpha )\\ & \;=3\lambda_{1,k}^{2}-2\lambda_{1,k}\alpha \\ & \;\geq \frac{1}{4}|k{|}^{4/3},\\ |\lambda_{2,k}-\lambda_{3,k}{|}^{2} & \;=(\lambda_{1,k}-\alpha )(3\lambda_{1,k}+\alpha )\\ & \;\geq (\lambda_{1,k}-|\alpha |)(3\lambda_{1,k}-|\alpha |)\\ & \;\geq \left(2^{-(1/3)}-{\left(\frac{1}{2}\right)}^{2/3}\right)\left(3\cdot 2^{-(1/3)}-{\left(\frac{1}{2}\right)}^{2/3}\right)|k{|}^{4/3}\\ & \;\geq \frac{1}{4}|k{|}^{4/3}. \end{array}$$

Since $\lambda_{3,k}$ is the complex conjugate of $\lambda_{2,k}$ we also get

$$|\lambda_{1,k}-\lambda_{3,k}{|}^{2}\geq \frac{1}{2}|k{|}^{4/3}.$$

Then, for i,j,l∈{1,2,3} with i≠j≠l it follows that

3.21 $$\left|\frac{1}{\left(\lambda_{i,k}-\lambda_{j,k})(\lambda_{i,k}-\lambda_{l,k}\right)}\right|\leq \frac{4}{|k{|}^{4/3}}.$$

We will now proceed to estimate the coefficients of the initial data appearing in the numerators of $c_{1,k},c_{2,k},c_{3,k}$. By (3.16) we get $|\lambda_{1,k}|\leq 2^{1/3}|k{|}^{2/3}\leq 2|k{|}^{2/3}$. Hence, together with (3.18) we obtain for i,j∈{1,2,3} with i≠j for the coefficient in front of $T_{\text{in, k}}$ in (3.8)

3.22 $$|\lambda_{i,k}\lambda_{j,k}|\leq 4|k{|}^{4/3},$$

and for the coefficients in front of $T_{t,\mathrm{in},k}$ in (3.8) we get with the help of the triangle inequality

3.23 $$|\lambda_{i,k}+\lambda_{j,k}|\leq 4|k{|}^{2/3}.$$

With the previous estimates we are now in the position to conclude the proof of the lemma. For this purpose we will determine the lower bound for $c_{1,k}$. From the definition of $c_{1,k}$ given in (3.8) and the estimates (3.21)–(3.23) with i=1,j=2,l=3 we get

$$\begin{array}{rl} |c_{1,k}| & \;=\left|\frac{T_{tt,\mathrm{in},k}-(\lambda_{3,k}+\lambda_{2,k})T_{t,\mathrm{in},k}+\lambda_{2,k}\lambda_{3,k}T_{\mathrm{in},k}}{\left(\lambda_{1,k}-\lambda_{2,k})(\lambda_{1,k}-\lambda_{3,k}\right)}\right|\\ & \;\leq \frac{\max\{1,|\lambda_{2,k}\lambda_{3,k}|,|\lambda_{2,k}+\lambda_{3,k}|\}}{|(\lambda_{1,k}-\lambda_{2,k})(\lambda_{1,k}-\lambda_{3,k})|}\max\{||T_{\mathrm{in}}|{|}_{G_{\sigma }^{3/2}},||T_{t,\mathrm{in}}|{|}_{G_{\sigma }^{3/2}},||T_{tt,\mathrm{in}}|{|}_{G_{\sigma }^{3/2}}\}e^{-\sigma |k{|}^{2/3}}\\ & \;\leq C\max\{||T_{\mathrm{in}}|{|}_{G_{\sigma }^{3/2}},||T_{t,\mathrm{in}}|{|}_{G_{\sigma }^{3/2}},||T_{tt,\mathrm{in}}|{|}_{G_{\sigma }^{3/2}}\}e^{-\sigma |k{|}^{2/3}}. \end{array}$$

Using the symmetric structure of the estimates (3.21)–(3.23) for i,j,l∈{1,2,3} we obtain the same estimates for $|c_{2,k}|$ and $|c_{3,k}|$ and hence the assertion.

**Remark 3.7.**

In the proof of theorem 3.2 we have determined an exact form for the Fourier coefficients $T_{k}(t)$ of the solution by solving the ordinary differential equation (3.5). More precisely, we solve the ordinary differential equation with the support of the characteristic equation, i.e. with the roots $\lambda_{1,k}$, $\lambda_{2,k}$, $\lambda_{3,k}$ of the polynomial $p(\lambda )=\lambda^{3}-\alpha \lambda^{2}-k^{2}$. Lemma 3.4 exploits specifically the behaviour of these roots with increasing frequencies (eventually translating this behaviour as regularities of our solutions). A natural question is whether we might obtain an explicit form of the roots $\lambda_{1,k}$, $\lambda_{2,k}$, $\lambda_{3,k}$.

Since the polynomial p(λ) is of degree 3, we can indeed determine explicitly the values of $\lambda_{1,k}$, $\lambda_{2,k}$, $\lambda_{3,k}$. For the sake of completeness, we state here the exact values of the roots, that can be determined making use of the Cardano’s formula: for any α, k∈Z satisfying $k^{2}>-(1/27)\alpha^{3}$ (a relation which is fulfilled by frequencies $|k|>2{\left(1+|\alpha |\right)}^{3/2}$), we have the following expressions:

$$\begin{array}{rl} \lambda_{1,k} & \;=\frac{\alpha }{3}+t_{1}^{1/3}+t_{2}^{1/3},\\ \lambda_{2,k} & \;=\frac{\alpha }{3}-\frac{t_{1}^{1/3}+t_{2}^{1/3}}{2}+i\frac{t_{1}^{1/3}-t_{2}^{1/3}}{2}\sqrt{3}\\ \mathrm{and}\;\lambda_{3,k} & \;=\frac{\alpha }{3}-\frac{t_{1}^{1/3}+t_{2}^{1/3}}{2}-i\frac{t_{1}^{1/3}-t_{2}^{1/3}}{2}\sqrt{3}, \end{array}$$

where $t_{1,k}$ and $t_{2,k}$ are given by

$$t_{1/2,k}=\frac{-q}{2}\pm \sqrt{\frac{q^{2}}{4}+\frac{p^{3}}{27}},$$

with

$$p=-\frac{\alpha^{2}}{3},\;q=-\frac{2\alpha^{3}}{27}-k^{2}.$$

Despite this explicit formula, the obtained information is the same as in lemma 3.4: at high frequencies the leading terms of the above roots have dispersion of order $\pm |k{|}^{2/3}$, as |k|≫1.

## 4. Sobolev regularities and conclusion

We conclude our analysis with some final remarks about the well and ill-posedness of the linearized model (3.1) within lower regularities than the Gevrey-class 3/2 (such as Sobolev).

In theorem 3.2, we showed that any initial data with regularity Gevrey-class 3/2 generates a local-in-time classical solution of the linearized equation (3.1). It is natural to question whether this choice of function space and the exponent 3/2 are simply mathematical artefacts resulting from our analysis techniques, or if they actually represent an optimal framework for the well-posedness of the model. Our analysis suggests that Gevrey-class 3/2 is indeed an optimal choice, when we do not impose additional structural assumptions on the initial data. Although detailed calculations are omitted here, we can provide some heuristics to support this argument.

In (3.6), we determined an explicit formula for each mode $T_{k}(t)$ of the solution $T(x,t)=\sum_{k\in Z}T_{k}(t){\text{e}}^{\text{i}kx}$. Notably, $T_{k}(t)$ contains a factor of the form $c_{1,k}{\text{e}}^{\lambda_{1,k}t}$, which represents the leading term contributing to the regularity of T. The root $\lambda_{1,k}$ is real and behaves as $\lambda_{1,k}∼|k{|}^{2/3}$ at high frequencies |k|≫1. This insight is particularly relevant when considering initial data with Sobolev regularity or more generally, any Gevrey class m with m>3/2.

For Sobolev regularities, the modes $T_{k}(0)=T_{\text{in, k}}$ of the initial data and the coefficients $c_{1,k}$, $c_{2,k}$, $c_{3,k}$ decay polynomially as k→±∞. Consequently, the leading term $c_{1,k}{\text{e}}^{\lambda_{1,k}t}$ experiences rapid exponential growth in time of the form ${\text{e}}^{|k{|}^{2/3}t}$. As a result, the modes lose their polynomial decay property in k∈Z, which is characteristic of Sobolev regularity, and exhibit an exponential inflation in frequencies.

Due to the exponential growth of the modes $T_{k}(t)$, a solution T with initial data in Sobolev spaces (or in Gevrey classes m with m>3/2) would quickly escape these function spaces and enter the regime of so-called ultradistributions, namely the dual space of analytic functions. Specifically, the exponential inflation of frequencies in the Fourier series of T leads to a loss of an ‘infinite amount’ of derivatives, as the smoothness of the solution rapidly deteriorates. This strong instability prevents the well-posedness of (3.1) in the Sobolev setting or any Gevrey class m with m>3/2.

These aspects hold true when we do not impose any structural assumption on the initial data. On the other hand, we remark that the factor $c_{1,k}{\text{e}}^{\lambda_{1,k}t}$ generating the instabilities vanishes when the coefficient $c_{1,k}$ is identically null. In this setting, the remaining terms $c_{2,k}{\text{e}}^{\lambda_{2,k}t}$ and $c_{3,k}{\text{e}}^{\lambda_{3,k}t}$ in (3.6) are somehow harmless for the Sobolev regularity, since they decay exponentially in the frequencies (as the real parts of $\lambda_{2,k}$ and $\lambda_{3,k}$ are negative), producing a smoothing effect on the solution T. Imposing $c_{1,k}=0$ seems nevertheless a sort of technical assumption, since from (3.8) it would imply that

$$T_{tt,\mathrm{in},k}=(\lambda_{3,k}+\lambda_{2,k})T_{t,\mathrm{in},k}-\lambda_{2,k}\lambda_{3,k}T_{\mathrm{in},k}.$$

Furthermore, from lemma 3.4 we may write this relation just in terms of $\lambda_{1,k}$ and α, by means of

$$\lambda_{3,k}+\lambda_{2,k}=-\lambda_{1,k}-\alpha$$

and

$$\lambda_{2,k}\lambda_{3,k}={(\frac{\lambda_{1,k}-\alpha }{2})}^{2}+(\lambda_{1,k}-\alpha )(3\lambda_{1,k}+\alpha )=\frac{13}{4}\lambda_{1,k}^{2}-\frac{5}{2}\alpha \lambda_{1,k}-\frac{3}{4}\alpha^{2},$$

which together with remark 3.7 imply

$$T_{tt,\mathrm{in},k}+(\lambda_{1,k}+\alpha )T_{t,\mathrm{in},k}+(\frac{13}{4}\lambda_{1,k}^{2}-\frac{5}{2}\alpha \lambda_{1,k}-\frac{3}{4}\alpha^{2})T_{\mathrm{in},k}=0$$

and

$$\lambda_{1,k}=\frac{4}{3}\alpha +{(\frac{\alpha^{3}}{27}+\frac{k^{2}}{2}+\sqrt{\frac{\alpha^{3}k^{2}}{27}+\frac{k^{4}}{4}})}^{1/3}+{(\frac{\alpha^{3}}{27}+\frac{k^{2}}{2}-\sqrt{\frac{\alpha^{3}k^{2}}{27}+\frac{k^{4}}{4}})}^{1/3}.$$

Unfortunately, this relation seems to be too technical to have a meaningful physical interpretation. In the absence of this restriction, however, the considered linearized equation is ill-posed in Sobolev spaces and it is rather improbable that its nonlinear counterpart (2.5) presents better properties with the nonlinear terms stabilizing the overall solutions.

However, as we showed in theorem 3.2, local-in-time smooth solutions of the linearized model exist if the initial conditions belong to regular enough Gevrey spaces.

## Data Availability

This article has no additional data.
